# Supplementary material for: Epigenetic clock and DNA methylation analysis of porcine models of aging and obesity
Source: GeroScience. 2021 Sep 15;43(5):2467–83. doi: 10.1007/s11357-021-00439-6 (PMC8599541; doi:10.1007/s11357-021-00439-6)
Supplement: Supplementary file 1 — (DOCX 704 KB) [file 11357_2021_439_MOESM1_ESM.docx]

**Supplementary Material**

for "Epigenetic clock and DNA methylation analysis of porcine models of aging and obesity"

**Supplementary Figure 1**. Unsupervised hierarchical clustering of porcine tissues. Average linkage hierarchical clustering based on the interarray correlation coefficient (Pearson correlation). Contrasting the first color band (based on cluster branches) with the second color band shows that the arrays cluster by tissue type (blood=blue, bladder=turquoise, frontal brain cortex=brown, kidney=yellow, liver=green, lung=red). Pig line: turquoise=domestic, brown=Wisconsin Miniature Swine, blue= Domestic/Minnesota Mini Cross. The last color encodes sex (pink=female).


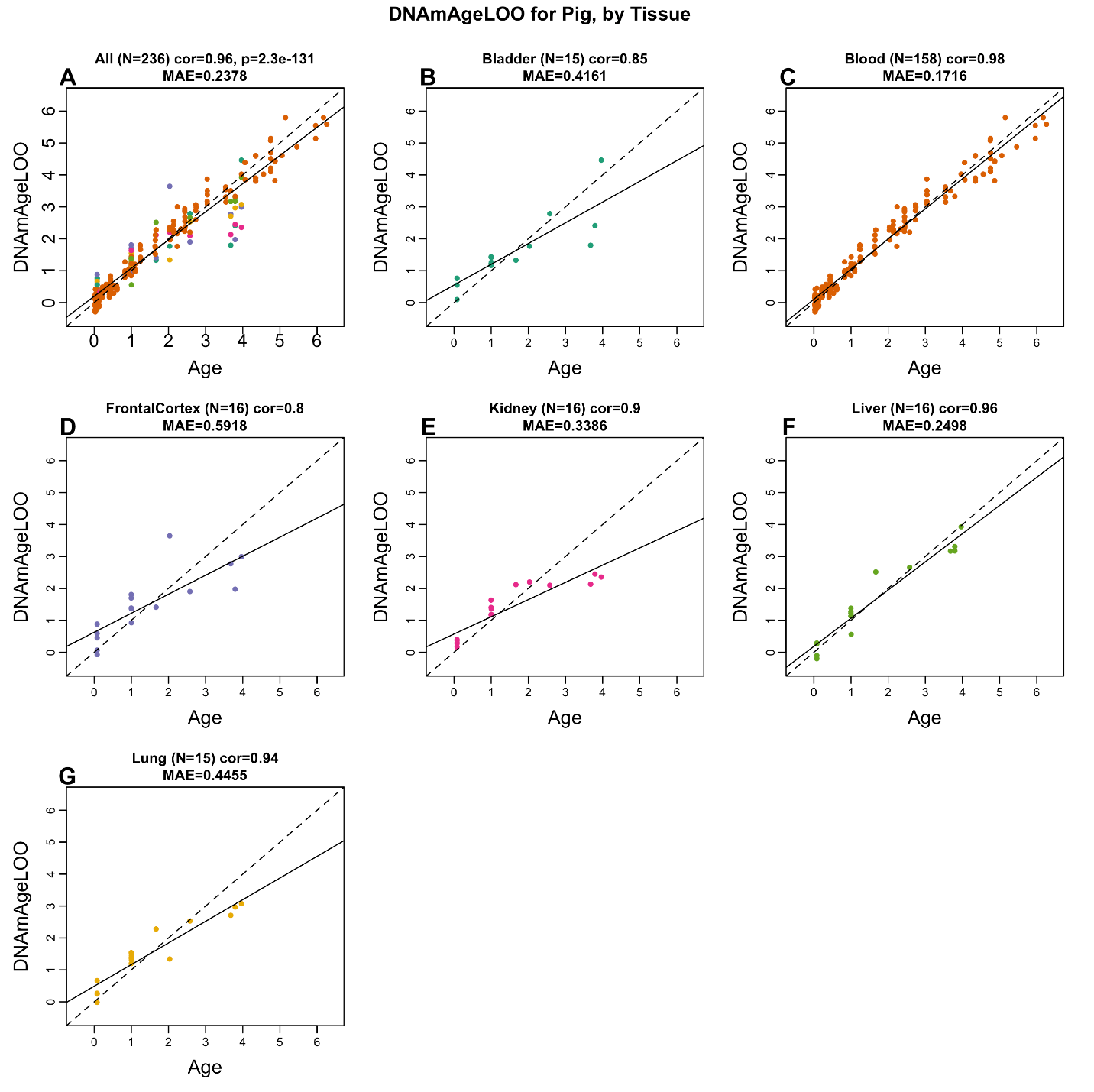


**Supplementary Figure 2. Pan tissue clock for pigs applied to individual tissues**. A) All porcine tissues combined. B) Bladder, C) Blood, D) Frontal cortex (brain), E) Kidney, F) Liver, G) Lung. Each panel reports the leave-one-out estimate of age (y-axis) versus chronological age (in years). Each title reports the sample size, Pearson correlation coefficient, and median absolute error.


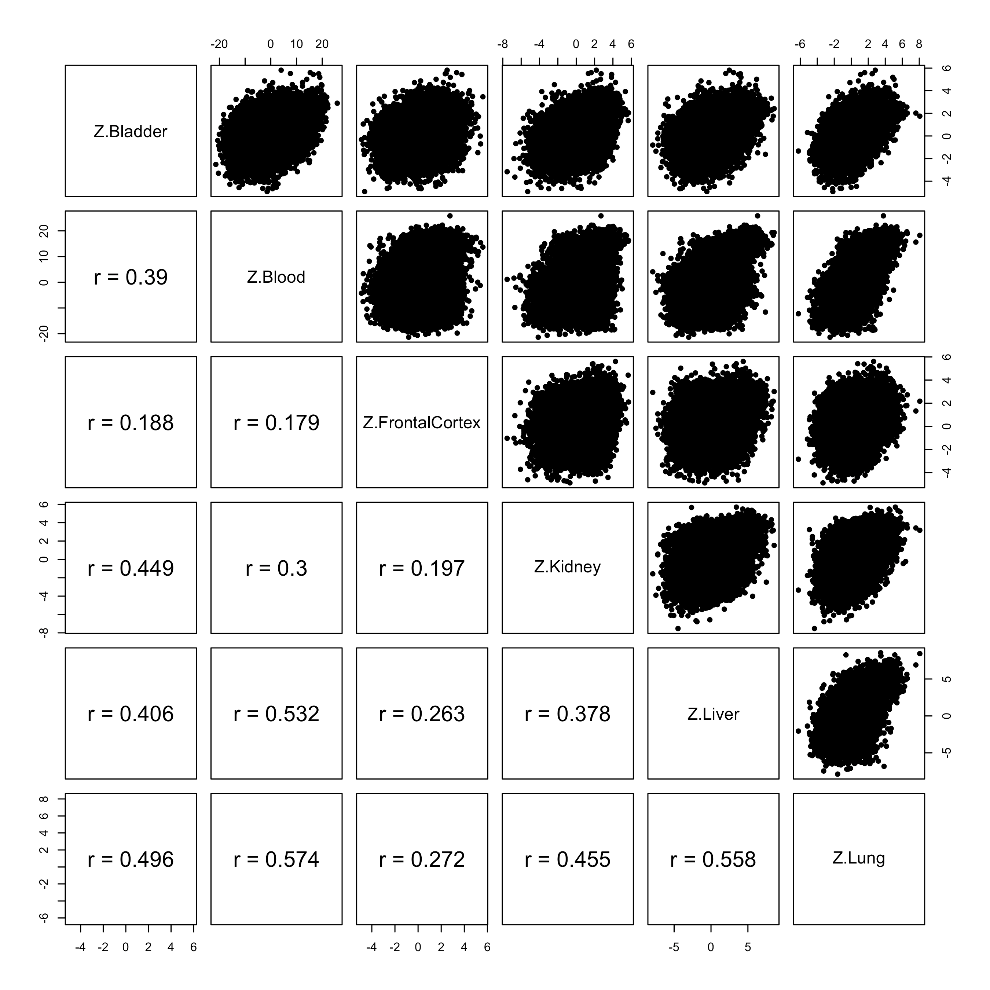


**Supplementary Figure 3**. Epigenome wide association study of correlation in different tissues. Each dot corresponds to a CpG. Z statistics for a correlation test of age in the respective tissues.


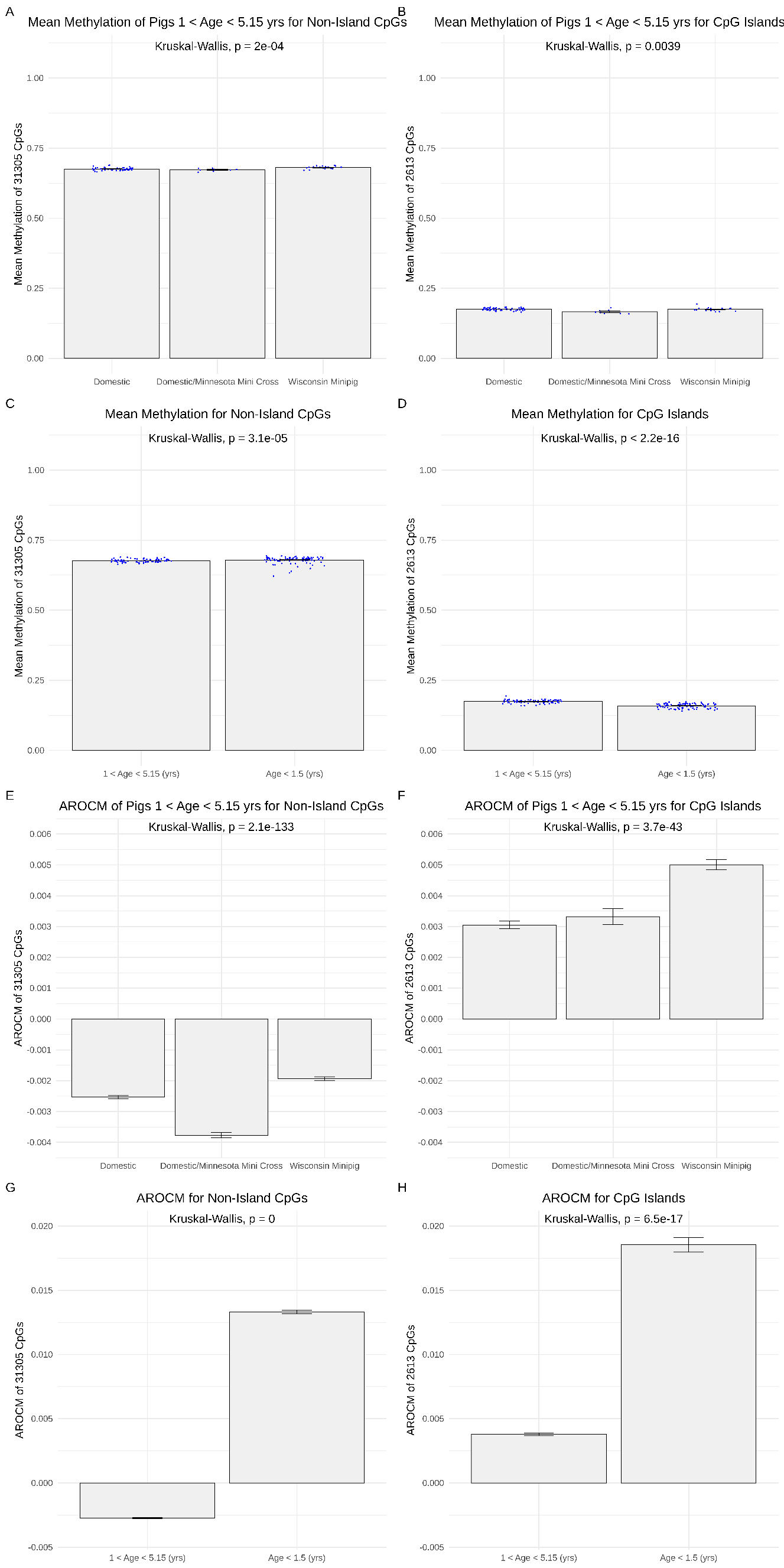


**Supplementary Figure 4. Mean Methylation and Average Rate of Change of Methylation**

A, B, C, D) Mean methylation of all CpG sites that (A, C) map to the Sus scrofa genome on a custom mammalian methylation array and (B, D) occur only within CpG islands. Figures (A) and (B) contain samples between the ages of 1 and 5.15 years old and are grouped by breed. Figures (C) and (D) group samples into two age ranges: between 1 and 5.15 years old, and less than 1.5 years old. Blood samples were taken from three breeds: “Domestic” pigs, a cross between “Domestic” and “Minnesota Mini” pigs, and “Wisconsin Minipigs”. Each point represents the mean methylation of all selected CpG sites in a single sample. The standard error was calculated using the standard deviation of the average methylation across the individual samples.

E,F) Average rate of change of methylation (AROCM) of all CpG sites that (E) map to the Sus scrofa genome on a custom mammalian methylation array and (F) occur only within CpG islands. Figures (E) and (F) contain samples between the ages of 1 and 5.15 years old and are grouped by breedBlood samples were taken from three breeds: “Domestic” pigs, a cross between “Domestic” and “Minnesota Mini” pigs, and “Wisconsin Minipigs”. AROCM is defined for each breed as the average of the rates of change at the selected CpG sites. The rate of change at each CpG is the slope coefficient of a simple linear regression where the beta values of a single CpG are regressed on the ages of the samples. The standard error was calculated using the standard deviation of the rate of change of methylation across the selected CpG sites.

To ensure a balanced statistical design, we restricted the analysis to pigs aged between 1 and 5.2. The title of each figure reports a non-parametric group comparison test (Kruskal Wallis tests).
